# Recent Highlights in Polymer Analysis and Characterization

**DOI:** 10.3390/polym17212846

**Published:** 2025-10-25

**Authors:** Giulio Malucelli

**Affiliations:** Department of Applied Science and Technology, Local INSTM Unit, Viale Teresa Michel 5, 15121 Alessandria, Italy; giulio.malucelli@polito.it; Tel.: +390131229369

Dear colleagues and friends,

The Polymer Analysis and Characterization Section has published high-quality Special Issues and scientific papers on various topics in polymer analysis and characterization.

Of the published papers, a few stand out, and I would like to draw your attention to them.

Some of these papers address fascinating aspects of additive manufacturing technologies. In this context, Farkas et al. [1] evaluated the influence of printing layer direction and thickness on the tensile and compression properties of a digital light processing 3D-printable dental material. Regardless of the direction of printing or layer thickness, tensile tests carried out on the obtained specimens revealed brittle behavior. In addition, the highest tensile values were measured for specimens printed with a 0.05 mm layer thickness.

Zhen et al. [2] thoroughly investigated how experimental parameters such as extrusion rate, filling angle, and printing orientation influence the mechanical behavior of 3D-printed poly(ether-ether-ketone) parts. Additionally, they tuned both crystallinity and strength through the application of various heat treatment conditions. The best mechanical performance was found to be attained at 1.0 times the extrusion rate, with varied angle cross-fillings within ±10°, and with vertical printing. Conversely, horizontal printing resulted in reduced warpage.

Guessasma and co-workers [3] used the DLP method to create 3D-printed blends of a photocurable, acrylate-modified poly(lactic acid) with lignin extracted from softwoods at loadings of 5–30 wt.%. They found that a post-curing treatment was useful for modulating the brittleness of the printed parts.

Ashebir et al. [4] critically discussed manufacturing defects in fiber-reinforced thermoplastic composites and classified fused filament fabrication-induced defects based on their morphology, location, and size. They also reviewed advanced non-destructive testing techniques, such as micro-computed tomography and structural health monitoring systems integrated with self-sensing fibers. Additionally, this paper highlighted the role of machine learning algorithms in enhancing the sensitivity and reliability of non-destructive testing methods, demonstrating that machine learning integration can improve defect detection by up to 25–30% compared to traditional techniques.

Zisopol et al. [5] developed a multi-objective optimization approach to determine the optimal 3D printing parameters (layer thickness and infill percentage) for efficiently producing poly(lactic acid) and acrylonitrile–butadiene–styrene parts. They optimized the balance between use value and production cost using a value analysis method. Infill percentage significantly influenced the ratio of use value to production cost for tensile, compression, and hardness tests, while flexural tests were affected by layer thickness. Impact strength was influenced almost equally by both factors, with material-specific variations.

The Polymer Analysis and Characterization Section also benefited from interesting contributions addressing the end of polymer systems’ lifespans. Some examples are summarized below.

Jones et al. [6] discussed the variability in the thermomechanical behavior of virgin and recycled polypropylene/high-density polyethylene blends when no other components were added. Differential scanning calorimetry revealed that both the recycled and virgin blends are immiscible. Generally, recycled blends showed lower overall crystallinity and melting temperatures than virgin blends, though their crystallization temperatures were comparable. Dynamic mechanical analysis revealed minimal variation in the storage modulus of recycled and virgin blends. However, the alpha and beta relaxation temperatures were lower in recycled blends due to structural deterioration. Additionally, the tensile properties of the recycled blends were affected by the recycling process. Young’s modulus and the yield strength of the recycled blends were lower than those of the virgin blends due to deterioration during the recycling process. However, the ductility of recycled blends was higher than that of virgin blends, possibly due to the plasticizing effect of low-molecular-weight chain fragments.

Han et al. [7] conducted a thorough review of the research on waste tire pyrolysis, including the pyrolysis mechanism, important factors affecting pyrolysis (e.g., temperature and catalysts), and the composition, properties, and applications of the three types of pyrolysis products.

Borelbach et al. [8] studied the degradation behavior of fibers made from poly(lactic acid) and its blend with poly(hydroxyalkanoate), as well as a bicomponent fiber made from poly(butylene succinate) and poly(lactic acid). The fibers were stored in topsoil at 23 °C for 12 weeks and in compost at 58 °C for four weeks (the latter to investigate degradation in an industrial composting plant). After 12 weeks in soil at ambient temperatures, neither the poly(lactic acid) nor the bicomponent fibers showed signs of degradation. However, the poly(hydroxyalkanoate)-based fibers exhibited cracking, as revealed by scanning electron microscopy, a decrease in molecular weight, and changes in the infrared spectrum. Under industrial composting conditions, the strength and molecular weight of all fibers decreased.

Other interesting papers investigated some specific topics related to the analysis and characterization of polymer systems.

Khaleel Ibrahim and Movahedi Rad [9] studied the plastic behavior of strengthened haunched beams using carbon fiber-reinforced polymers. They utilized a probabilistic design that took into account random concrete properties, carbon fiber-reinforced polymer properties, and complementary strain energy values. The reliability index was used as a limiting index because the proposed method considered probabilistic parameters for models with limited plastic behavior, which are designed based on the reliability index. As the reliability index value increased and the corresponding load value decreased, the load and deflection values were affected by variable randomness. This indicated an increased probability of failure in models subjected to higher loading conditions. The results showed that as the produced load increased, so did the intensity of the damage.

Cona et al. [10] reviewed methods for the chemical, physical, and mechanical characterization of interpenetrating polymer networks (IPNs) to detect their formation and develop hydrogel properties. The researchers also evaluated the effectiveness of these methods in confirming the formation of IPNs.

Jung [11] developed four effective, complementary methods for measuring gas absorption uptake from polymers enriched with pure gas under high pressure and for determining gas diffusivity. These methods included the gravimetric method, the volumetric and gas chromatography methods, and the manometric method, which are based on mass, volume, and pressure measurements, respectively. Representative results of the methods, like gas uptake, solubility, and diffusivity, were demonstrated. The measuring principles, procedures, and characteristics of the methods were compared, as were the measured results.

Cruz-Morales et al. [12] reviewed the synthesis, structure, and properties of natural and synthetic rubber, particularly the synthesis of polyisoprene rubber nanocomposites. Readers were provided with a comprehensive reference on how to mimic natural rubber.

All of the aforementioned research outcomes demonstrate the remarkable research activities carried out in the Polymer Analysis and Characterization Section of *Polymers*.

With the help of the editorial board, editorial staff, and esteemed readers and authors, this Section is expected to continue growing and expanding in 2026!

## Data Availability

Dataset available on request from the author.
